# Evaluation of Integrated HPV DNA as Individualized Biomarkers for the Detection of Recurrent CIN2/3 during Post-Treatment Surveillance

**DOI:** 10.3390/cancers13133309

**Published:** 2021-07-01

**Authors:** Heike Hoyer, Grit Mehlhorn, Cornelia Scheungraber, Ingke Hagemann, Christine Hirchenhain, Linn Woelber, Claudia Stolte, Monika Hampl, Sarah Scherbring, Agnieszka Denecke, Janina Bartels, Andreas D. Ebert, Sabina Meneder, Annett Petzold, Tabitha Heller, Karsten R. Heidtke, Elisabeth Schwarz, Frederik Stübs, Stefanie Schütze, Eva-Lena Stange, Anna Jaeger, Franca Martignoni, Ansgar Dellmann, Achim Rody, Peter Hillemanns, Tanja Fehm, Karl-Ulrich Petry, Gerd Böhmer, Barbara Schmalfeldt, Pauline Wimberger, Matthias W. Beckmann, Ingo B. Runnebaum, Matthias Dürst

**Affiliations:** 1 Institut für Medizinische Statistik, Informatik und Datenwissenschaften, Universitätsklinikum Jena, 07743 Jena, Germany; heike.hoyer@med.uni-jena.de; 2 Frauenklinik, Universitätsklinikum Erlangen, 91054 Erlangen, Germany; grit.mehlhorn@uk-erlangen.de (G.M.); frederik.stuebs@uk-erlangen.de (F.S.); fk-direktion@uk-erlangen.de (M.W.B.); 3 Klinik und Poliklinik für Frauenheilkunde und Fortpflanzungsmedizin, Universitätsklinikum Jena, 07747 Jena, Germany; scheungraber@outlook.com (C.S.); annett.petzold@med.uni-jena.de (A.P.); stefanie.schuetze@med.uni-jena.de (S.S.); ingo.runnebaum@med.uni-jena.de (I.B.R.); 4 abts+partner Partnerschaftsgesellschaft, Prüner Gang 7, 24103 Kiel, Germany; i.hagemann@abts-partner.de (I.H.); eva-lena.stange@posteo.de (E.-L.S.); 5 Klinik und Poliklinik für Frauenheilkunde und Geburtshilfe, Technische Universität Dresden, 01307 Dresden, Germany; christine.hirchenhain@uniklinikum-dresden.de (C.H.); pauline.wimberger@uniklinikum-dresden.de (P.W.); 6 Klinik für Gynäkologie, Universitätsklinikum Hamburg-Eppendorf, 20246 Hamburg, Germany; lwoelber@uke.de (L.W.); a.jaeger@uke.de (A.J.); b.schmalfeldt@uke.de (B.S.); 7 Institut für Zytologie und Dysplasie (IZD), Theaterstr. 14, 30159 Hannover, Germany; stolte@izd-hannover.de (C.S.); boehmer@izd-hannover.de (G.B.); 8 Frauenklinik, Universitätsklinikum Düsseldorf, 40225 Düsseldorf, Germany; hampl@med.uni-duesseldorf.de (M.H.); frauenarzt-westend@medicover.de (F.M.); tanja.fehm@med.uni-duesseldorf.de (T.F.); 9 Fachärzte für Frauenheilkunde und Geburtshilfe, Karrenführerstraße 1-3, 38100 Braunschweig, Germany; info@gyn-braunschweig.de; 10 Klinikum Wolfsburg, 38440 Wolfsburg, Germany; agnieszka.denecke@klinikum.wolfsburg.de (A.D.); gyn@klinikum.wolfsburg.de (K.-U.P.); 11 Klinik für Frauenheilkunde und Geburtshilfe, Medizinische Hochschule Hannover (MHH), 30625 Hannover, Germany; bartels@kinderwunsch-hildesheim.de (J.B.); hillemanns.peter@mh-hannover.de (P.H.); 12 Praxis für Gynäkologie und Geburtshilfe, Ärztehaus Nürnberger Str. 67, 10787 Berlin, Germany; info@prof-ebert.de; 13 Klinik für Frauenheilkunde und Geburtshilfe, Universitätsklinikum Schleswig-Holstein (UKSH), 23538 Lübeck, Germany; sabina.meneder@uksh.de (S.M.); achim.rody@uksh.de (A.R.); 14 Zentrum für Klinische Studien (ZKS), Universitätsklinikum Jena, 07747 Jena, Germany; tabitha.heller@med.uni-jena.de; 15 ATLAS Biolabs GmbH, Aroser Allee 68, 13407 Berlin, Germany; heidtke@atlas-biolabs.com; 16 Deutsches Krebsforschungszentrum, ATV, 69120 Heidelberg, Germany; elisabeth.schwarz@alumni.dkfz.de; 17 Städtisches Klinikum Braunschweig gGmbH, 38118 Braunschweig, Germany; a.dellmann@klinikum-braunschweig.de

**Keywords:** HPV DNA integration, CIN3, recurrence, post treatment surveillance, individualized molecular biomarker

## Abstract

**Simple Summary:**

HPV-DNA integration into the host genome is a frequent step in cervical carcinogenesis and is considered to be a tumor-driving event. Viral integration sites are unique for each patient and could thus serve as highly specific molecular markers for the detection of recurrent disease. Unexpectedly, our study showed that integration sites as individualized biomarkers could not detect all recurrent pre-cancers (CIN2/3). Nevertheless, this is the first study which has identified and validated integration sites in a large number of CIN3 (*n* = 445) and as such has unraveled several novel findings: The integration frequency observed in CIN3 was much lower than anticipated (10.8% in CIN3 vs. 80% in cervical cancer). Moreover, in contrast to the monoclonal situation in cervical carcinoma, integrated HPV-DNA in CIN3 is most likely confined to clonally expanding subpopulations.

**Abstract:**

Purpose: Post-treatment follow-up in women with cervical pre-cancers (CIN3) is mandatory due to relapse in up to 10% of patients. Standard follow-up based on hrHPV-DNA/cytology co-testing has high sensitivity but limited specificity. The aim of our prospective, multicenter, observational study was to test the hypothesis that an individualized viral-cellular-junction test (vcj-PCR) combined with cytology has a lower false positive rate for the prediction of recurrence compared to standard co-testing. Methods: Pre-surgical cervical swabs served for the identification of HPV16/18 DNA integration sites by next-generation-sequencing (NGS). Samples taken at 6, 12 and 24 months post-surgery were evaluated by cytology, hrHPV-DNA and the patients’ individual HPV-integration sites (vcj-PCR on the basis of NGS). Results: Integration sites were detected in 48 of 445 patients (10.8%), 39 of them had valid follow-up data. The false positive rate was 18.2% (95% CI 8.6–34.4%) for standard hrHPV/cytology at six months compared to 12.1% (95% CI 4.8–27.3%) for vcj-PCR/cytology, respectively (McNemar *p* = 0.50). Six patients developed recurrences (1 CIN2, 5 CIN3) during follow-up. Standard co-testing detected all, whereas vcj-PCR/cytology detected only five patients with recurrences. Data of 269 patients without evidence of HPV16/18 integration were subject to post-hoc analyses. Standard co-testing revealed a false positive rate of 15.7% (95% CI 11.7–20.7%) and predicted ten of fourteen recurrences at six months. Conclusions: Although highly specific on its own vcj-PCR could not detect all recurrent CIN2/3. Possible reasons for this unexpected result may be multifocal lesions, intratumoral heterogeneity with respect to HPV integration and/or incident CIN.

## 1. Introduction

Prophylactic vaccination to prevent infection by high-risk human papillomaviruses (hrHPV) combined with cervical cancer screening as a secondary prevention step is the best strategy to minimize the number of deaths caused by cervical cancer. Ideally screening aims to identify high grade cervical precursor lesions (cervical intraepithelial neoplasia Grade 2 (CIN2) and Grade 3 (CIN3)) and early stage cervical cancers. Treatment of CIN2/3 is achieved by loop electrosurgical excision procedure (LEEP) or ablation to prevent progression to cervical cancer. Despite treatment, around 10% of patients develop recurrent high-grade disease within two years of follow-up [1,2,3]. Recurrent disease is twofold in nature, representing either persisting lesions resulting from residual disease or incident (new) disease. Most of the recurrent disease diagnosed within two years of surgery is actually residual disease [4]. There are several factors that influence recurrent disease: (1) Persistence with HPV16 is associated with a greater rate of relapse after conisation of CIN2+ with respect to the other hrHPV genotypes [5,6]. (2) For women with adenocarcinoma in situ (AIS) age ˃ 30 years, positive endocervical margins and lesions ˃ 8 mm were associated with a higher risk of AIS recurrence [7]. (3) Although the pathologic margin status is generally considered to be a risk factor for the development of recurrent CIN, a free margin does not always indicate complete excision because of the possibilities of multifocal lesions or inadequate specimen tissue due to ablative conisation [8]. In view of the substantial risk of recurrent disease effective post-treatment testing is mandatory. In a systematic review and meta-analysis Kocken and colleagues compared hrHPV-testing, cytology and hrHPV/cytology co-testing in predicting post-treatment disease after conisation. The best result in terms of sensitivity (95%, 95% CI 91–98%), albeit at the expense of specificity (67%, 95% CI 60–74%), was achieved by co-testing 6 months after treatment [9]. An algorithm comprising co-testing after 6, 12 and 24 months is now standard of care in many countries including Germany [10,11,12].

Next to hrHPV/cytology co-testing the performance of several other markers for the monitoring of women treated for high grade CIN is being explored. Post-treatment monitoring by CADM1/MAL-methylation analysis identified women with an increased risk of recurrent CIN2/3 [13]. Moreover, a significant increase in specificity for recurrent disease was observed for hrHPV/p16/Ki67/co-testing in comparison to hrHPV/cytology co-testing [14,15].

In terms of individualized molecular markers for post-treatment monitoring HPV DNA integration is of interest. HPV DNA integration into the host genome is characteristic for most cervical carcinomas [16,17,18,19,20,21]. Moreover, HPV integration sites were shown to be unique for each patient [22,23,24,25]. By an innovative technology which combines target enrichment and next generation sequencing (TEN16/18) HPV DNA integration sites can be readily identified [24]. This DNA sequence information permits PCR-primer design to validate the integration site by a viral-cellular junction PCR (vcj-PCR). Recently, we could show by vcj-PCR that 7 of 8 cervical carcinomas are homogenous with respect to intra-tumoral distribution of the integration sites indicative of clonal expansion [26]. There are also several reports in the literature indicating that HPV DNA integration may also be a decisive step for progression within the CIN spectrum [18,23,27]. Because of the uniqueness of the HPV integration sites the primary objective of our study was to show that co-testing by an individualized viral-cellular-junction test (vcj-PCR) and cytology has superior specificity for the prediction of recurrent disease during post-surgery surveillance compared to standard co-testing by a hrHPV test and cytology. Unexpectedly, although highly specific on its own, vcj-PCR could not detect all recurrent CIN2/3.

## 2. Materials and Methods

### 2.1. Study Design

**Figure 1:**
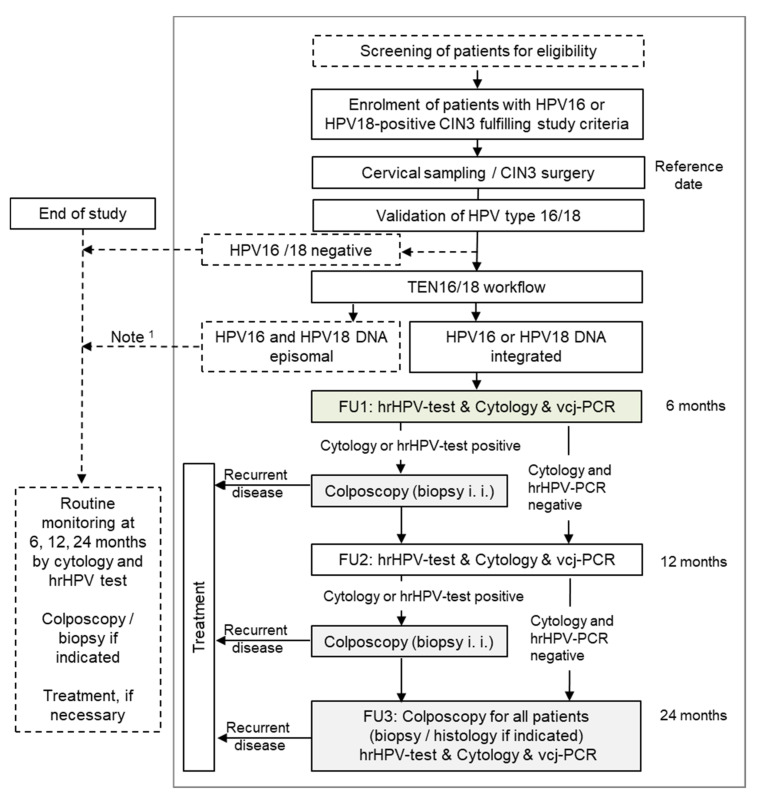
Flow chart of study procedures according to the study protocol. HPV—human papilloma virus; CIN3—cervical intraepithelial neoplasia Grade 3; TEN—target enrichment and next generation sequencing; FU—follow-up visit; hrHPV—high risk HPV; vcj-PCR—viral cellular junction polymerase chain reaction; i. i.—if indicated; ^1^ end of study was intended for patients without evidence of HPV16/18 integration. Because the TEN16/18 analyses required more time than initially anticipated, a subset of patients without evidence of HPV-DNA integration were followed further by standard co-testing with HPV test and cytology within the study. Their data was used for post-hoc analyses.

We performed a two-step, multicenter, prospective observational study in the target setting of monitoring patients after CIN3 surgery (Figure 1). The study was registered in the German Clinical Trials Register (DRKS00010435). We evaluated the diagnostic accuracy of two co-testing methods in the same subjects against the same reference procedure (paired design). Patients with histologically confirmed HPV16 or 18 positive CIN3 were eligible if they underwent surgery for the first time, were aged at least 18 years and had given informed consent. Re-conisation was an exclusion criterion. Gynecologists from twelve German colposcopy clinics enrolled patients. Before surgery a study specific cervical swab was taken and sent to the study laboratory to validate HPV 16 or 18 infection. The sample was then processed further to identify HPV 16/18 DNA integration sites and to develop an individualized vcj-PCR assay (Supplementary Figure S1). As recommended by the German monitoring guidelines three follow-up exams were scheduled at six, twelve and twenty four months after surgery. Patients routinely underwent co-testing by cytology and hrHPV test. From patients with evidence of HPV 16/18 integration all study specific cervical swabs were evaluated by vcj-PCR. In this subgroup of patients we compared the performance of two monitoring strategies to predict recurrent disease: (i) cytology and hrHPV (standard) and (ii) cytology and vcj-PCR (new). This was the objective of study Step 2. Due to technical difficulties HPV16/18 integration status remained unknown longer than expected. Therefore, we followed-up all participants, who agreed, by standard and study-specific examinations. Thus, additional data on standard monitoring could be collected for patients without evidence of HPV16/18 integration at point of surgery. This subgroup was called post-hoc analysis population. Owing to the experimental character, vcj-PCR results of Step 2 participants were blinded during the study and medical decisions were made in accordance with the current standards of care.

Colposcopy was done at the discretion of the attending physician during the first and second follow-up visit, but was requested for all Step 2 participants at the last follow-up visit. Study data was recorded via customized electronic case report forms in two independent databases. One database comprised all data collected by the different study sites. The other one was used to record the vcj-data generated at Jena University Hospital (JUH) gynecology research laboratory. The data management staff from Center of Clinical Studies at JUH developed and maintained both databases. The study was approved by the institutional review board at JUH (vote: 4671-01/16) for the principal investigator and by the local review committees for all participating sites, respectively. Sex as a biological variable was no issue because of the nature of the disease investigated.

### 2.2. Identification of Viral-Cellular Junctions (vcj) and Development of the Individualized vcj-PCR

The identification of HPV integration sites was achieved by use of the TEN16 protocol developed by Xu and colleagues [24]. This protocol was modified to also identify HPV18 integration sites and was conducted by ATLAS Biolabs GmbH (Berlin, Germany). The major steps comprise DNA fragmentation and adapter tagging, PCR enrichment of the early viral region, Illumina next-generation sequencing and data processing. The starting material for the identification of viral-cellular junctions was DNA extracted from cervical swabs taken less than four weeks before or at the time point of surgery. In order to rule out experimental artefacts all candidate integration sites were validated by vcj-PCR. This was achieved by designing a cellular and a viral PCR primer pair unique for each junction on the basis of the NGS data (Supplementary Figure S1). Ideally the PCR product should be in the range of 150 to 300 bp. Typically, the vcj-PCR reaction mix in an end volume of 20 µL contained 20 ng DNA, 10 µL 2× Fast Start Universal SYBR Green Mix (Merck, Darmstadt, Germany) and 5pmol of each primer. After 10 min of initial denaturation at 95 °C, 10 cycles were performed for 30 s at 95 °C, 20 s at 68 °C (minus 1 °C per cycle) and 30 s at 72 °C, followed by 25 cycles each for 30 s at 95 °C, 20 s at 58 °C and 30 s at 72 °C. The reaction was finalized for 2 min at 72 °C. All PCR-reactions were analyzed in 2% agarose gels and vcj-PCR products of the expected size were subjected to Sanger sequencing to confirm the integration site as determined by the TEN16/18 protocol. To assure sufficient sensitivity of the vcj-PCR for the later application on cervical swabs during follow-up, a dilution series of the original DNA (initial visit) was evaluated. The vcj-PCR protocol was judged as being satisfactory if the viral-cellular junction fragment was detectable at a dilution of 10^−3^. Moreover, for a subset of patients the vcj-PCR results of all follow-up swabs were successfully replicated. All vcj-PCR were performed in the research laboratory of the Department of Gynecology at JUH.

### 2.3. Index Tests and Reference Procedures

The standard monitoring procedures cytology and hrHPV test were performed and analyzed at study sites according to the local standards. Conventional or thin-layer liquid-based cytology was applied. Using the Munich III nomenclature for Pap smear samples results were categorized as insufficient material (0), negative (I, II-a, II-p, II-g and II-e) or positive (≥III). This corresponds to the Bethesda system as follows: 0 = Unsatisfactory for evaluation, I and II-a = NILM, II-p = ASC-US, II-g = AGC endocervical NOS, II-e = endometrial cells, and ≥III-p, III-g, III-e, III-x = ASC-H, AGC or higher. For hrHPV testing the commercially available tests HC2, Cobas HPV test and the GenID HPV assay (AID Easy-Typing Kit) were used. Additionally, the JUH gynecology research laboratory analyzed hrHPV in study-specific cervical samples by the GP5+ and GP6+ bio assay [28]. By this assay 20 different genital HPV types, including all hrHPV types can be amplified. If the patient’s local hrHPV test was missed at one visit we used the respective hrHPV test result from the JUH gynecology research laboratory for the statistical analysis.

In terms of accuracy studies, cytology and vcj-PCR co-testing represented the experimental index test (new), cytology and hrHPV co-testing represented the comparator index test (standard). A co-test was designated positive if at least one of the two single tests was positive. Otherwise, the co-test was negative, even in the rare case of missing one of the two test results.

To detect recurrent disease gynecologists examined their patients by colposcopy. If indicated, they took a biopsy in accordance with local practice. Recurrent disease was diagnosed if suspected CIN2, CIN3 or cervical cancer (CIN2+) could be confirmed by histology. Thus, the reference procedure comprised colposcopy and histopathology, if appropriate.

### 2.4. Study Endpoints

The primary study endpoint was the false positive rate (FPR = 1-specificity) of an index test at first follow-up six months after surgery in patients with evidence of HPV16/18 integration. We defined the FPR as the proportion of test positives out of all patients without recurrent disease up to 24 months after CIN3 surgery. The true positive rate (TPR = sensitivity) was determined as the key secondary endpoint. TPR is equivalent to the proportion of test positives at first follow-up out of patients with recurrent disease at or after this visit. We defined predictive values and test positive rates as further secondary endpoints. All performance parameters had to be assessed for the index co-tests as well as for the single tests. Additionally, we specified the prevalence of primary CIN3 cases with evidence of HPV16/18 DNA integrates as a secondary endpoint.

### 2.5. Statistical Analysis

In study Step 1, we calculated the prevalence of HPV16/18 DNA integration with 95% confidence interval (CI). In study Step 2 we compared the predictive accuracy of co-testing by cytology and vcj-PCR (new) with co-testing by cytology and hrHPV-test (standard) in patients with evidence of HPV16/18 DNA integration. For co-tests and single tests performed six months after surgery we calculated true, false and test positive rates and predictive values for the prediction of recurrent disease using standard definitions. Moreover, for each test we calculated an overall false positive rate as the proportion of patients with at least one positive result during repeated follow-up visits in those without recurrent disease. Wilson 95% two-sided confidence intervals were estimated for proportions [29]. For the new co-test we hypothesized superiority with regard to the false positive rate (FPR). The ratio of false positive rates FPRnew/FPRstandard was calculated with the two-sided 95% CI for a paired design as proposed by Alonzo and colleagues [30]. We applied the exact McNemar test for the paired comparison with a two-sided significance level of 5%. Non-inferiority of the new co-test was hypothesized for the true positive rate (TPR). Due to the small number of patients with evidence of HPV16/18 DNA integration and disease recurrence we report the frequency of true positives for single and co-tests and omit the statistical comparison.

Post-hoc analyses: Since follow-up data was available from a sufficient number of patients without evidence of integration, we additionally analyzed the performance of standard tests in these patients. Moreover, we compared the cumulative probability of recurrent disease in patients with and without integration by means of product-limit estimates and log-rank test. SAS 9.4 was used for statistical analyses.

A sample size of 300 patients with evidence of HPV16/18 DNA integration and valid data was planned for study Step 2. From the meta-analysis of Kocken and colleagues [9] we assumed for the standard co-testing a false positive rate of 33%, a true positive rate of 95% and a rate of disease recurrence up to 24 months after surgery of 10%. For the new co-test we expected a FPR of 10% and a TPR not worse than 85% (non-inferiority margin). For the paired comparisons we needed 76 patients without recurrent disease to show superiority (power 90%, alpha 2.5% one-sided) and 26 patients with recurrence to show non-inferiority (power 80%, alpha 5% one-sided) of the new co-test according to Alonso and colleagues [30]. Expecting a dropout rate of 10% and an integration rate of 50% we planned to enroll 670 patients into study Step 1 (Appendix A: sample size calculation).

## 3. Results

### 3.1. Recruitment and Patients Characteristics

**Figure 2:**
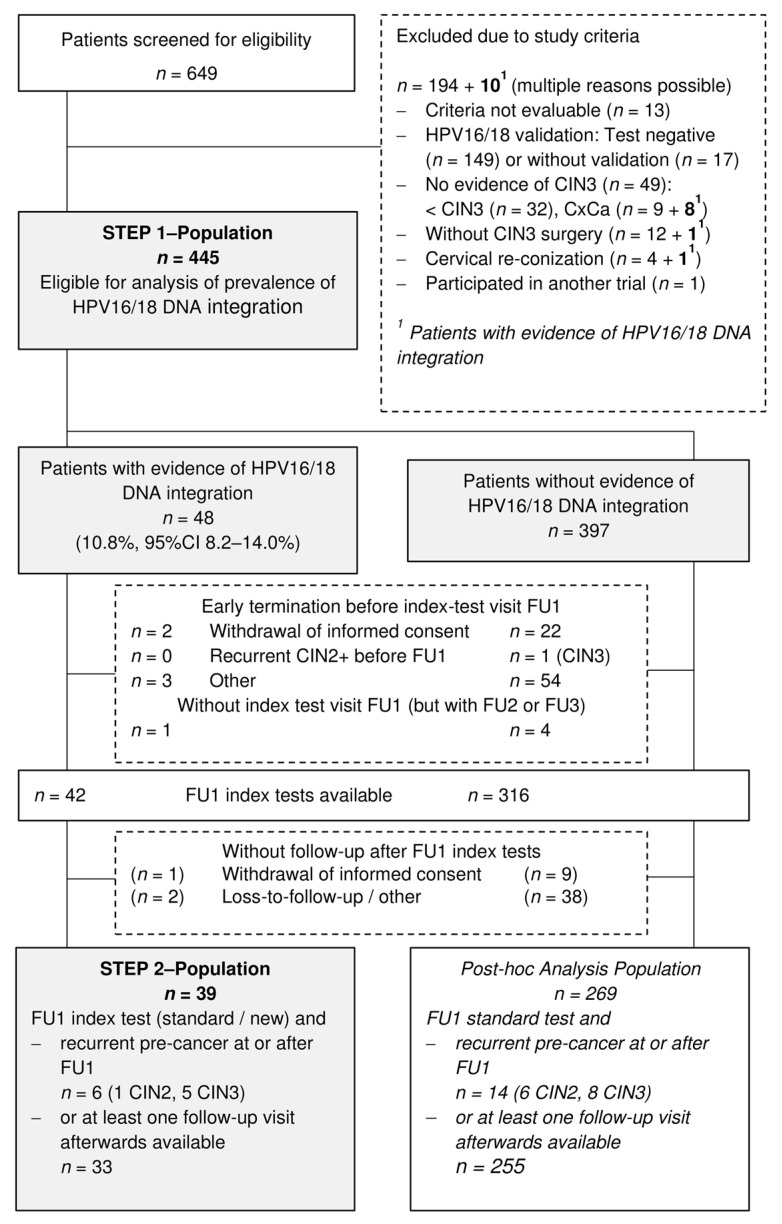
Flow diagram of the study population (HPV—human papilloma virus, CIN2/3—cervical Intraepithelial Neoplasia Grade 2/3, CxCa—cervical cancer, FU1/2/3—follow-up 1/2/3).

From May 2016 to September 2017, gynecologists from 12 German study sites screened 649 patients for eligibility to participate in our two-step prospective observational study (Figure 2).

Overall, 204 patients had to be excluded mainly for two reasons: (1) HPV16/18 infections could not be validated in cervical samples of 166 patients. These patients were screened predominantly at one site, where HPV genotyping was not routinely performed. (2) In 49 patients, CIN3 was not confirmed by histology. Among those excluded HPV16/18 DNA integration sites were identified in cervical samples of 10 patients. Eight of them were diagnosed with cervical cancer at surgery (Figure 2). Table 1 displays the characteristics of those 445 patients (age range 21 to 57 years), who were eligible for the analysis of integration frequency (Step 1). HPV16/18 DNA integration sites could be identified in the cervical swab of 48 patients taken prior to surgery resulting in an integration prevalence of 10.8% (95% CI 8.2–14.0%).

**Figure 3:**
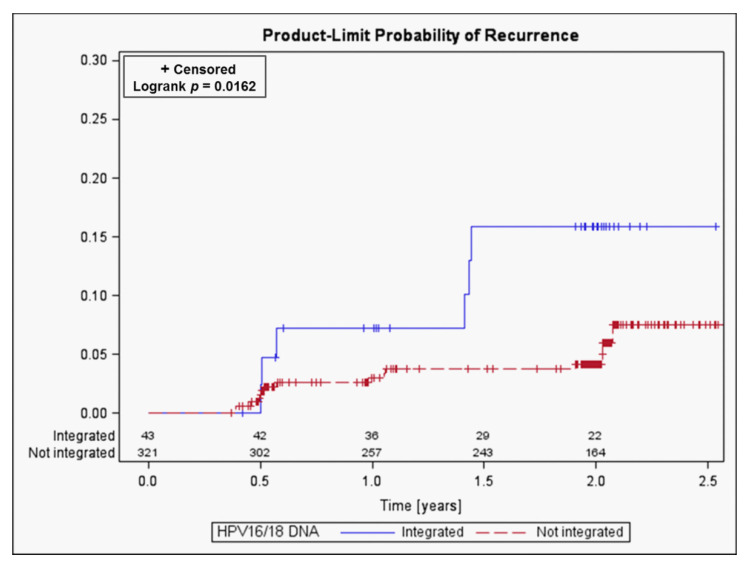
Cumulative probability of disease recurrence according to HPV16/18 DNA integration status.

Details of the integration sites with respect to viral break points, chromosomal location and cellular genes affected are summarized in Supplementary Table S1 and reflect the observations made for cervical cancers [24]. Eight of 48 patients harbored more than one integration site. Of the above 48 patients 42 attended at least the first follow-up visit (FU1) about six months after surgery for monitoring by cytology, hrHPV-test and vcj-PCR (index tests). Thirty-nine patients were followed over a median of two (range 0.5 to 2.5) years, five of them were missed at the last follow-up visit (Figure 2). They form the study population for the comparative analysis of index test performance (Step 2). Additionally, 269 patients without evidence of HPV16/18 DNA integration were eligible for post-hoc analyses. They were routinely monitored by cytology and hrHPV-test and could be followed over a median of two (range 0.4 to 3.0) years. Twenty-three patients did not attend the last follow-up visit. Overall, recurrent disease was detected in six patients with evidence of integration (one CIN2 and five CIN3 at or after FU1) and 15 patients without evidence of integration (one CIN3 before FU1, six CIN2 and eight CIN3 at or after FU1). Figure 2 shows the complete flow diagram. The cumulative probability of recurrent disease, presented in Figure 3, was significantly higher in patients with than without evidence of HPV16/18 DNA integration (15.9% vs. 7.5%, *p* = 0.016).

### 3.2. Performance of Index Tests in Patients with Evidence of HPV16/18 DNA Integration

Results of single tests and co-tests over three follow-up visits at six, twelve and twenty-four months after surgery are shown in the appendix (Table A1). Table 2 comprise true, false and test positive rates at the first follow-up visit (FU1) to predict recurrent disease at or after FU1 in patients with evidence of HPV16/18 DNA integration. In 33 patients without recurrence the false positive rate for the new co-test (vcj-PCR/cytology) was 12.1% (95% CI 4.8–27.3%) compared to 18.2% (95% CI 8.6–34.4%) for the standard co-test (hrHPV/cytology) (exact McNemar *p* = 0.5). We estimated a false positive rate ratio of 0.67. The 95% confidence interval of 0.38 to 1.17 covered one, the value of indifference. The new co-test missed one of six patients with recurrence whereas all recurrences were predicted correctly by the standard co-test. Vcj-PCR alone discovered three of six recurrences and was negative for all 33 patients without recurrence. Predictive values for FU1 index tests are summarized in Table 3. We calculated negative predictive values of 96.7% (95% CI 83.3–99.4%) and 100% (95% CI 87.5–100%) for the new and the standard co-test, respectively. Five of nine patients with a positive new co-test experienced recurrent disease compared to six of twelve patients with a positive standard co-test. The number of test positives drives the recall of patients. Here, 23.1% (95% CI 12.6–38.3%) would be recalled after FU1 by the new co-test and 30.8% (95% CI 18.6–46.4%) by the standard co-test, respectively (Table 2). From this the new to standard rate ratio of 0.75 (95% CI 0.54–1.04) resulted. Moreover, we summarized false positive test results over repeated follow-up visits. Out of patients without recurrence 15.2% (95% CI 6.7–30.9%) received at least one false positive result by the new co-test, 21.2% (95% CI 10.7–37.8%) by the standard co-test, but 0% (95% CI 0–10.4%) by vcj-PCR alone. We calculated an overall false positive rate ratio (new/standard) of 0.71 (95% CI 0.45–1.14).

### 3.3. Performance of Standard Tests in Patients without Evidence of HPV16/18 DNA Integration (Post-Hoc Analyses)

Table A2 in the appendix shows all test results that were available for 269 patients without evidence of HPV16/18 DNA integration at point of surgery. This population was monitored according to the standard follow-up for patients after CIN3 surgery. The standard co-test at first follow-up revealed a false positive rate of 15.7% (95% CI 11.7–20.7%) and a test positive rate of 18.6% (95% CI 14.4–23.7%) (Table 2). Ten of fourteen cases with recurrent disease were truly positive for the standard co-test at FU1. We estimated a positive predictive value of 20% (95% CI 11.2–33.0%) and a negative predictive value of 98.2% (95% CI 95.4–99.3%) (Table 3).

## 4. Discussion

### 4.1. HPV-DNA Integration in CIN3

The concept of this study was based on the assumption that at least 50% of CIN3 would harbor integrated HPV DNA [18]. Among the Step 1 population of our study (*n* = 445), only 10.8% harbored either HPV16 or HPV18 integrated DNA. To date, this is the largest number of CIN3 cases in which the physical state of the viral genome was analyzed by an NGS-based approach. Possibly Hu and colleagues [18] have overestimated the number of integration sites in their CIN cohort (*n* = 27) as not every candidate integration site identified by NGS was validated by PCR and Sanger sequencing [31]. Indeed, by use of the same technology as Hu and colleagues, Liu and colleagues reported an integration rate of only 26.7% (95% CI 7.8–55.1%) among 15 CIN3 cases [20]. Thus, the only modest integration rate we observed in CIN3 most probably reflects the true integration frequency. Of note is that eight of seventeen (47%) patients who had to be excluded from our study because of microinvasive disease in their cone had integrated HPV (Figure 2). In a previous study, by use of the very same TEN-technology an integration rate of 80% was observed in HPV16 positive cervical carcinoma biopsies [24]. This evident difference in the integration rate between CIN3 and cervical cancer supports the concept that tumor-driving integration events occur in a minority of high-grade CIN giving rise to clonal sub-populations with higher malignant potential. In the present study, multiple integration sites were identified in 6 of 39 (15%) of HPV16-positive cases and 2 of 9 (22%) of HPV18 cases. Of the six patients with integrated HPV DNA who developed recurrent CIN2/3 only 3 were detected by vcj-PCR during follow-up. Interestingly, these 3 patients were the ones with multiple integration sites. Moreover, during follow-up some of these integration sites were lost arguing against the presence of multiple integration sites within a single cell but rather for the presence of different clonal sub-populations. To rule out false negative results due to sampling error we performed short tandem repeats (STR) analyses for DNA from the initial visit and the corresponding follow-up visits for the 3 recurrent cases which were not detected by vcj-PCR. Moreover, sufficient sensitivity of the individual vcj-PCR assays was assured by performing dilution series of DNA from the initial visit. Detection was achieved at a dilution of 10^−3^.

### 4.2. Predictive Accuracy of vcj-PCR/Cytology Co-Testing Compared to the Standard Co-Testing

Overall, the study could not confirm our initial assumption that an individualized viral-cellular-junction test (vcj-PCR) combined with cytology should have a lower false positive rate and therefore superior specificity compared to standard co-testing for the prediction of recurrence (CIN2/3 or cervical cancer) within two years after CIN3 surgery. Among patients without recurrence the false positive rate for the new co-test (vcj-PCR/cytology) with 12.1% (95% CI 4.8–27.3%) 6 months after treatment was more favorable compared to 18.2% (95% CI 8.6–34.4%) for the standard co-test (hrHPV/cytology) but the difference was not statistically significant. It should be emphasized that these results are based on a thoroughly planned prospective, multicenter, observational study which adhered to the German guidelines for post-conisation surveillance. The cohort design was appropriate to prevent spectrum bias. The predictive accuracy was compared in a paired design where patients underwent both index tests. Study data was captured electronically and was managed in two quality-assured databases. The central study management supported and motivated the local staff. We performed on-site quality control at eleven of twelve study centers which enrolled at least five patients into study Step 1. Source data were checked for all patients with evidence of HPV16/18 DNA integration and for all patients with recurrent diseases. Concerning the limitations we largely failed to obtain the necessary sample size for the accuracy comparisons of false and true test positive rates. As discussed above, we assumed that an individual vcj-PCR test could be developed for about 50% of enrolled patients. However, we observed HPV16/18 DNA integration in only 10.8% of cervical samples at point of surgery. Taking into account all study criteria, data from only 39 patients with integration and follow-up data were available for index test comparisons. This sample size was clearly inadequate to evidence the postulated effects. Nevertheless, it was sufficient to demonstrate the limits of the proposed concept since three of six recurrent disease cases were missed by the sole vcj-PCR test. Second, for ethical reasons, we chose a reference procedure which is commonly used in this setting. Patients eligible for the accuracy study should undergo colposcopy at least at the third follow-up visit. If indicated by respective findings, a biopsy should be taken thereafter. Some patients did not attend the final visit. However, all but one were examined by colposcopy at least twice during follow-up. Thus, our reference procedure was similarly imperfect for both index tests performed for the same patient. We estimated the ratio of false positive rates as effect measure of comparative accuracy which is proposed in this case [32]. Third, results of cytology and hrHPV test at follow-up were interpreted locally at study sites without knowledge of the recurrent disease status. Vcj-PCR was performed independently of local tests and without knowledge of the recurrence status at the JUH research laboratory. However, study specific cervical samples from each visit were also tested for hrHPV by the JUH research laboratory. Hence, the patient’s hrHPV status was not masked to the research laboratory team.

### 4.3. Post Hoc Analyses of Patients without HPV16/18 DNA Integration

Because the TEN16/18 analyses required more time than initially anticipated, 269 patients without evidence of HPV-DNA integration were available for post-hoc analyses. Based on this population several highly relevant observations were made. Among the above patients, 14 developed recurrent CIN2/3 at or after the first follow-up visit. HPV-testing and cytology (co-testing) at 6 months post conisation (FU1) was negative for 4 of these patients. Sensitivity with 71.4% was thus clearly lower than the 95% (95% CI 91–98%) presented in the meta-analysis of Kocken and colleagues [9]. On the other hand, the false positive rate was more favorable in our cohort with 15.7% (95% CI 11.7–20.7%) vs. 33% (95% CI 26–40%). Of note is that of the four patients who were negative by standard co-testing at 6 months (FU1) three tested positive at FU2 (one year) and one at FU3 (two years). Thus, in our cohort (including the patients with integrated HPV-DNA) all recurrences were detected by the standard co-testing within the recommended follow-up time of 24 months.

We also observed that the cumulative probability of recurrent disease (Figure 3) was significantly higher in patients with than without evidence of HPV16/18 DNA integration (15.9% vs. 7.5%, *p* = 0.016). This unexpected finding suggests that remnants of dysplastic cell clusters with integrated HPV DNA may have a higher potential for recurrence. However, this is not in line with the observation that only 3 of the 6 recurrences could be detected by vcj-PCR. It may thus be speculated that the HPV-DNA integration status as such reflects the genetic instability of the entire HPV-infected epithelium thereby paving the way for recurrence. Future studies will show whether HPV DNA integration in CIN may serve as a prognostic factor for progression and recurrence.

## 5. Conclusions

This large prospective, multicenter, observational study clearly demonstrated that the individualized viral-cellular-junction test (vcj-PCR) combined with cytology had a somewhat lower false-positive rate than the standard hrHPV/cytology co-testing for the detection of recurrent disease but lacked sensitivity, and therefore, poses no alternative for the current post-treatment surveillance. Moreover, even in case of superiority, the observed low integration frequency would preclude implementation of the vcj-PCR approach in a clinical setting. Nevertheless, the study has provided valuable novel insights into the complex issue of HPV-DNA integration and cervical carcinogenesis and should stimulate further investigation. In particular, the low integration frequency observed in CIN3 as compared to cervical cancer (10.8% vs. 80%) indicates that HPV integration is a late event in carcinogenesis. Moreover, integrated HPV-DNA in CIN3 is most likely confined to clonally expanding subpopulations which may explain the deficiencies of vcj-PCR as an individualized marker for detecting recurrent disease. Other explanations for the low sensitivity observed would be multifocal lesions and/or incident CIN.

## Figures and Tables

**Table 1:** Characteristics of patients according to the HPV16/18 DNA integration status (Step 1, analysis population).

Patient Characteristics	with Integration (*n* = 48)	without Integration (*n* = 397)	Total (*n* = 445)
Age (years), median (range)	33 (23–51)	32 (21–57)	32 (21–57)
Number of life births, *n* [%]			
None	24 (50.0)	193 (48.6)	217 (48.8)
One	13 (27.1)	113 (28.5)	126 (28.3)
More than one	11 (22.9)	91 (22.9)	102 (22.9)
Any contraception, *n* [%]	34 (70.8)	251 (63.4)	285 (64.2)
Thereof contraceptive pill	23 (67.6)	162 (64.5)	185 (64.9)
Thereof condom	4 (11.8)	52 (20.7)	56 (19.6)
Pregnant at study entry, *n* [%]	0	2 (0.5)	2 (0.4)
Menopause, *n* [%]	0	11 (2.8)	11 (2.5)
Smoking status, *n* [%]			
Current smoker	19 (39.6)	180 (45.3)	199 (44.7)
Ex-smoker	7 (14.6)	42 (10.6)	49 (11.0)
Non-smoker	21 (43.7)	168 (42.3)	189 (42.5)
Unknown	1 (2.1)	7 (1.8)	8 (1.8)
HPV vaccination, *n* [%]	5 (12.8)	46 (13.7)	51 (13.6)
Type of primary surgery, *n* [%]			
High-frequency (HF) loop	33 (68.8)	282 (71.0)	315 (70.8)
Laser conisation	10 (20.8)	75 (18.9)	85 (19.1)
HF loop and laser conisation	5 (10.4)	40 (10.1)	45 (10.1)
Resection margin, *n* [%]			
Negative	30 (62.5)	266 (67.0)	296 (66.5)
Positive	8 (16.7)	52 (13.1)	60 (13.5)
Unknown	10 (20.8)	79 (19.9)	89 (20.0)

**Table 2:** True, false and test positive rates of first follow-up tests in patients with and without HPV16/18 DNA integration to predict recurrent pre-cancer at visit or afterwards.

First Follow-Up 6 Months after Surgery	True Positive Rate ^(a)^	False Positive Rate 95% CI [%]			Test Positive Rate 95% CI [%]		
With integration (Step 2 analysis population, *n* = 39)							
Cytology	(4/6)	(4/32)	12.5	5.0–28.1	(8/38)	21.1	11.1–36.3
hr-HPV	(5/6)	(3/33)	9.1	3.1–23.6	(8/39)	20.5	10.8–35.5
vcj-PCR	(3/6)	(0/33)	0.0	0.0–10.4	(3/39)	7.7	2.7–20.3
Cytology + vcj-PCR (new)	(5/6)	(4/33)	12.1	4.8–27.3	(9/39)	23.1	12.6–38.3
Cytology + hrHPV (standard)	(6/6)	(6/33)	18.2	8.6–34.4	(12/39)	30.8	18.6–46.4
Rate ratio (new/standard)			0.67	0.38–1.17		0.75	0.54–1.04
Without integration (post-hoc analysis population, *n* = 269)							
Cytology	(5/13)	(16/242)	6.6	4.1–10.5	(21/255)	8.2	5.4–12.3
hr-HPV	(9/14)	(28/255)	11.0	7.7–15.4	(37/269)	13.8	10.1–18.4
Cytology + hrHPV (standard)	(10/14)	(40/255)	15.7	11.7–20.7	(50/269)	18.6	14.4–23.7

^(a)^ Due to low number percentage is not presented.

**Table 3:** Positive and negative predictive values of first follow-up tests in patients with and without HPV16/18 DNA integration to predict recurrent pre-cancer at visit or afterwards.

First Follow-Up 6 Months after Surgery	Positive Predictive Value 95% CI [%]			Negative Predictive Value 95% CI [%]		
With integration (Step 2, analysis population, *n* = 39)						
Cytology	(4/8)	^(a)^		(28/30)	93.3	78.7–98.2
hr-HPV	(5/8)	^(a)^		(30/31)	96.8	83.8–99.4
vcj-PCR	(3/3)	^(a)^		(33/36)	91.7	78.2–97.1
Cytology + vcj-PCR (new)	(5/9)	^(a)^		(29/30)	96.7	83.3–99.4
Cytology + hr-HPV (standard)	(6/12)	^(a)^		(27/27)	100	87.5–100
Without integration (post-hoc analysis population, *n* = 269)						
Cytology	(5/21)	23.8	10.6–45.1	(226/234)	96.6	93.4–98.3
hr-HPV	(9/37)	24.3	13.4–40.1	(227/232)	97.8	95.1–99.1
Cytology + hr-HPV (standard)	(10/50)	20.0	11.2–33.0	(215/219)	98.2	95.4–99.3

^(a)^ Due to low number percentage is not presented.

## Data Availability

Sequence data of all viral-cellular DNA junctions were deposited in BankIt of the NCBI database https://www.ncbi.nlm.nih.gov/ (accessed on 11 May 2021).

## Supplementary Materials

The following are available online at https://www.mdpi.com/article/10.3390/cancers13133309/s1, Figure S1: schematic of study work flow, Table S1: viral-cellular junctions of all patients validated by vcj-PCR.

## Appendix A

### Sample Size Calculation

We wanted to compare the accuracy of co-testing by cytology and vcj-PCR (new) with co-testing by cytology and hrHPV-test (standard). For the new test we hypothesized superiority with regard to the false positive rate FPR (1-specificity) which was the primary endpoint and non-inferiority of true positive rate TPR (sensitivity) which was the key secondary endpoint. From the meta-analysis of Kocken et al. [9] we assumed a TPR_standard_ of 95%, a FPRs_tandard_ of 33% and a rate of disease recurrence up to 24 months after surgery of 10%.

For statistical comparison we planned to use the relative false positive rate rFPR_new/standard_ (=FPR_new_/FPR_standard_) and the relative true positive rate rTPR_new/standard_ (=TPR_new_/TPR_standard_), respectively. Inference should be based on confidence intervals (CI) of relative true and relative false positive rate for a paired design as proposed by Alonzo et al. [30]. Thus, the same approach was considered for superiority and non-inferiority hypothesis testing.

Justification of sample size for testing the non-inferiority hypothesis of the true positive rate (key secondary endpoint): First, we calculated the number of patients with recurrence needed to show non-inferiority of TPR_new_. The expected TPR_new_ was 95% (equal to standard) and the expected rTPR_new/standard_ was 1. For the proof of concept, TPR_new_ ≤ 85% was considered as inferior resulting in a non-inferiority margin for rTPR_new/standard_ of 0.89 (0.85/0.95):

Null hypothesis      H0: rTPR_new/standard_ ≤ δTPR = 0.89,

Alternative hypothesis   HA: rTPR_new/standard_ > δTPR = 0.89.

Additionally, the sample size formula [30] for a paired-design required specification of the proportion of positive results in both tests. Here, we assumed 92.5%, the mean of possible values under the alterative hypothesis. To ensure a reasonable but feasible number of patients with recurrence in our study, power of 80% and one-sided alpha of 5% was considered for the non-inferiority test of TPR as secondary endpoint. Under these assumptions non-inferiority of TPR_new_ would be shown with power of 80% if data of 26 patients with recurrent diseases could be analyzed. Taking into account sampling variation, a total cohort size of 300 patients with HPV16/18 DNA integration was planned for the comparison of accuracy. If recurrence rate was 10%, we would observe at least 26 recurrent cases with probability of 80% in this cohort.

Justification of sample size for testing the superiority hypothesis of the false positive rate (primary endpoint): To show superiority of FPR_new_ we assumed a clinically relevant FPR_new_ of 10% resulting in rFPR_new/standard_ of 0.30 (0.10/0.33):

Null hypothesis      H0: rFPR_new/standard_ ≥ δFPR = 1.0,

Alternative hypothesis   HA: rFPR_new/standard_ < δFPR = 1.0.

The proportion of positive results in both tests was assumed to be 5% (mean of possible values under the alterative hypothesis). We would need 74 patients without recurrence to show superiority of the FPR_new_ with power of 90% and one-sided alpha of 2.5. Thus, a total cohort size of 300 patients with HPV16/18 DNA integration would always be sufficient to test the superiority hypothesis for the false positive rate.

Total number of patients to be enrolled: expecting a dropout rate of 10%, 334 patients with evidence of HPV16/18 DNA integration would be needed for the accuracy study. With regard to the integration rate of 50%, 670 patients should enroll.

**Table A1:** Test results in patients with HPV16/18 DNA integration according to recurrent disease (CIN2+) at visit or afterwards (Step 2 analysis population). Patients with recurrence finished study participation after detection of CIN2+ during follow-up and were treated according to current German Guidelines.

Predictive Tests	Patients with HPV16/18 DNA Integration (*n* = 39)						
	with Recurrent Pre-Cancer at or after Visit			without Signs of Recurrence at or after Visit			without Visit
First Follow-up	*n* = 6 (4 before second follow-up)			*n* = 33			*n* = 0
Test results ^1^, *n*	Positive	Negative	Missing	Positive	Negative	Missing	
Cytology	4	2		4	28	1	
hr-HPV	5	1		3	30		
vcj-PCR	3	3		0	33		
Cytology + hr-HPV (standard)	6	0		6	27		
Cytology + vcj-PCR (new)	5	1		4	29		
Second follow-up	*n* = 2 (2 before third follow-up)			*n* = 33			*n* = 0
Test results ^1^, *n*	Positive	Negative	Missing	Positive	Negative	Missing	
Cytology	1	0	1	2	27	4	
hr-HPV	2	0		1	31	1	
vcj-PCR	1	1		0	32	1	
Cytology + hr-HPV (standard)	2	0		3	29	1	
Cytology + vcj-PCR (new)	2	0		2	30	1	
Third follow-up	*n* = 0			*n* = 28			*n* = 5
Test results ^1^, *n*	Positive	Negative	Missing	Positive	Negative	Missing	
Cytology				1	26	1	5
hr-HPV				0	27	1	5
vcj-PCR				0	27	1	5
Cytology + hr-HPV (standard)				1	27	0	5
Cytology + vcj-PCR (new)				1	27	0	5

^1^ None of the tests revealed indeterminate results, the missing category shows the number of missing tests.

**Table A2:** Test results in patients without HPV16/18 DNA integration according to recurrent disease (CIN2+) at visit or afterwards (post-hoc analysis population). Patients with recurrence finished study participation after detection of CIN2+ during follow-up and were treated according to current German Guidelines.

Predictive Tests	Patients without HPV16/18 DNA Integration (*n* = 269)						
	with Recurrent Pre-Cancer at or after Visit			without Signs of Recurrence at or after Visit			without Visit
First Follow-up	*n* = 14 (7 before second follow-up)			*n* = 255			*n* = 0
Test results ^1^, *n*	Positive	Negative	Missing	Positive	Negative	Missing	
Cytology	5	8	1	16	226	13	
hr-HPV	9	5		28	227		
Cytology + hr-HPV (standard)	10	4		40	215		
Second follow-up	*n* = 7 (4 before third follow-up)			*n* = 255			*n* = 0
Test results ^1^, *n*	Positive	Negative	Missing	Positive	Negative	Missing	
Cytology	2	4	1	12	206	37	
hr-HPV	5	2		24	207	24	
Cytology + hr-HPV (standard)	6	1		35	196	24	
Third follow-up	*n* = 3 (3 at third follow-up)			*n* = 232			*n* = 23
Test results ^1^, *n*	Positive	Negative	Missing	Positive	Negative	Missing	
Cytology	2	1		11	215	6	23
hr-HPV	2	1		25	207		23
Cytology + hr-HPV (standard)	3	0		34	198		23

^1^ None of the tests revealed indeterminate results, the missing category shows the number of missing tests.

## References

[1] Arbyn M., Sasieni P., Meijer C.J., Clavel C., Koliopoulos G., Dillner J. (2006). Chapter 9: Clinical applications of hpv testing: A summary of meta-analyses. Vaccine.

[2] Lili E., Chatzistamatiou K., Kalpaktsidou-Vakiani A., Moysiadis T., Agorastos T. (2018). Low recurrence rate of high-grade cervical intraepithelial neoplasia after successful excision and routine colposcopy during follow-up. Medicine.

[3] Ghaem-Maghami S., Sagi S., Majeed G., Soutter W.P. (2007). Incomplete excision of cervical intraepithelial neoplasia and risk of treatment failure: A meta-analysis. Lancet Oncol..

[4] Bjornerem M.S., Sorbye S.W., Skjeldestad F.E. (2020). Recurrent disease after treatment for cervical intraepithelial neoplasia-the importance of a flawless definition of residual disease and length of follow-up. Eur. J. Obstet. Gynecol. Reprod. Biol..

[5] Friebe K., Klapdor R., Hillemanns P., Jentschke M. (2017). The value of partial hpv genotyping after conization of cervical dysplasias. Geburtshilfe Frauenheilkd..

[6] Bruno M.T., Cassaro N., Garofalo S., Boemi S. (2019). Hpv16 persistent infection and recurrent disease after leep. Virol. J..

[7] Codde E., Munro A., Stewart C., Spilsbury K., Bowen S., Codde J., Steel N., Leung Y., Tan J., Salfinger S.G. (2018). Risk of persistent or recurrent cervical neoplasia in patients with ‘pure’ adenocarcinoma-in-situ (ais) or mixed ais and high-grade cervical squamous neoplasia (cervical intra-epithelial neoplasia grades 2 and 3 (cin 2/3)): A population-based study. BJOG Int. J. Obstet. Gynaecol..

[8] Kang W.D., Oh M.J., Kim S.M., Nam J.H., Park C.S., Choi H.S. (2010). Significance of human papillomavirus genotyping with high-grade cervical intraepithelial neoplasia treated by a loop electrosurgical excision procedure. Am. J. Obstet. Gynecol..

[9] Kocken M., Uijterwaal M.H., de Vries A.L., Berkhof J., Ket J.C., Helmerhorst T.J., Meijer C.J. (2012). High-risk human papillomavirus testing versus cytology in predicting post-treatment disease in women treated for high-grade cervical disease: A systematic review and meta-analysis. Gynecol. Oncol..

[10] Massad L.S., Einstein M.H., Huh W.K., Katki H.A., Kinney W.K., Schiffman M., Solomon D., Wentzensen N., Lawson H.W. (2013). 2012 updated consensus guidelines for the management of abnormal cervical cancer screening tests and cancer precursors. Obstet. Gynecol..

[11] Munro A., Codde J., Semmens J., Leung Y., Spilsbury K., Williams V., Steel N., Cohen P., Pavicic H., Westoby V. (2015). Utilisation of co-testing (human papillomavirus DNA testing and cervical cytology) after treatment of cin: A survey of gps’ awareness and knowledge. Aust. Fam. Physician.

[12] S3-Leitlinie Prävention des Zervixkarzinoms. AWMF Registernummer: 015/027OL..

[13] Uijterwaal M.H., van Zummeren M., Kocken M., Luttmer R., Berkhof J., Witte B.I., van Baal W.M., Graziosi G.C.M., Verheijen R.H.M., Helmerhorst T.J.M. (2016). Performance of cadm1/mal-methylation analysis for monitoring of women treated for high-grade cin. Gynecol. Oncol..

[14] Liu W., Gong J., Xu H., Zhang D., Xia N., Li H., Song K., Lv T., Chen Y., Diao Y. (2020). Good performance of p16/ki-67 dual-stain cytology for detection and post-treatment surveillance of high-grade cin/vain in a prospective, cross-sectional study. Diagn. Cytopathol..

[15] Polman N.J., Uijterwaal M.H., Witte B.I., Berkhof J., van Kemenade F.J., Spruijt J.W., van Baal W.M., Graziosi P.G., van Dijken D.K., Verheijen R.H. (2017). Good performance of p16/ki-67 dual-stained cytology for surveillance of women treated for high-grade cin. Int. J. Cancer.

[16] Durst M., Kleinheinz A., Hotz M., Gissmann L. (1985). The physical state of human papillomavirus type 16 DNA in benign and malignant genital tumours. J. Gen. Virol..

[17] Luft F., Klaes R., Nees M., Durst M., Heilmann V., Melsheimer P., von Knebel Doeberitz M. (2001). Detection of integrated papillomavirus sequences by ligation-mediated pcr (dips-pcr) and molecular characterization in cervical cancer cells. Int. J. Cancer.

[18] Hu Z., Zhu D., Wang W., Li W., Jia W., Zeng X., Ding W., Yu L., Wang X., Wang L. (2015). Genome-wide profiling of hpv integration in cervical cancer identifies clustered genomic hot spots and a potential microhomology-mediated integration mechanism. Nat. Genet..

[19] Akagi K., Li J., Broutian T.R., Padilla-Nash H., Xiao W., Jiang B., Rocco J.W., Teknos T.N., Kumar B., Wangsa D. (2014). Genome-wide analysis of hpv integration in human cancers reveals recurrent, focal genomic instability. Genome Res..

[20] Liu Y., Zhang C., Gao W., Wang L., Pan Y., Gao Y., Lu Z., Ke Y. (2016). Genome-wide profiling of the human papillomavirus DNA integration in cervical intraepithelial neoplasia and normal cervical epithelium by hpv capture technology. Sci. Rep..

[21] Durst M., Croce C.M., Gissmann L., Schwarz E., Huebner K. (1987). Papillomavirus sequences integrate near cellular oncogenes in some cervical carcinomas. Proc. Natl. Acad. Sci. USA.

[22] Schmitz M., Driesch C., Jansen L., Runnebaum I.B., Durst M. (2012). Non-random integration of the hpv genome in cervical cancer. PLoS ONE.

[23] Schmitz M., Driesch C., Beer-Grondke K., Jansen L., Runnebaum I.B., Durst M. (2012). Loss of gene function as a consequence of human papillomavirus DNA integration. Int. J. Cancer.

[24] Xu B., Chotewutmontri S., Wolf S., Klos U., Schmitz M., Durst M., Schwarz E. (2013). Multiplex identification of human papillomavirus 16 DNA integration sites in cervical carcinomas. PLoS ONE.

[25] Kraus I., Driesch C., Vinokurova S., Hovig E., Schneider A., von Knebel Doeberitz M., Durst M. (2008). The majority of viral-cellular fusion transcripts in cervical carcinomas cotranscribe cellular sequences of known or predicted genes. Cancer Res..

[26] Carow K., Golitz M., Wolf M., Hafner N., Jansen L., Hoyer H., Schwarz E., Runnebaum I.B., Durst M. (2017). Viral-cellular DNA junctions as molecular markers for assessing intra-tumor heterogeneity in cervical cancer and for the detection of circulating tumor DNA. Int. J. Mol. Sci..

[27] Woodman C.B., Collins S.I., Young L.S. (2007). The natural history of cervical hpv infection: Unresolved issues. Nat. Rev..

[28] Jacobs M.V., Snijders P.J., van den Brule A.J., Helmerhorst T.J., Meijer C.J., Walboomers J.M. (1997). A general primer gp5+/gp6(+)-mediated pcr-enzyme immunoassay method for rapid detection of 14 high-risk and 6 low-risk human papillomavirus genotypes in cervical scrapings. J. Clin. Microbiol..

[29] Newcombe R.G. (1998). Two-sided confidence intervals for the single proportion: Comparison of seven methods. Stat. Med..

[30] Alonzo T.A., Pepe M.S., Moskowitz C.S. (2002). Sample size calculations for comparative studies of medical tests for detecting presence of disease. Stat. Med..

[31] Dyer N., Young L., Ott S. (2016). Artifacts in the data of hu et al. Nat. Genet..

[32] Bossuyt P.M., Irwig L., Craig J., Glasziou P. (2006). Comparative accuracy: Assessing new tests against existing diagnostic pathways. BMJ.

