# Supplementary material for: Evaluation of Integrated HPV DNA as Individualized Biomarkers for the Detection of Recurrent CIN2/3 during Post-Treatment Surveillance
Source: Cancers (Basel). 2021 Jul 1;13(13):3309. doi: 10.3390/cancers13133309 (PMC8269020; doi:10.3390/cancers13133309)
Supplement: Supplementary file 1 [file cancers-13-03309-s001.zip › Supplementary Figure S1.pdf]

Figure S1: Schematic of study work flow

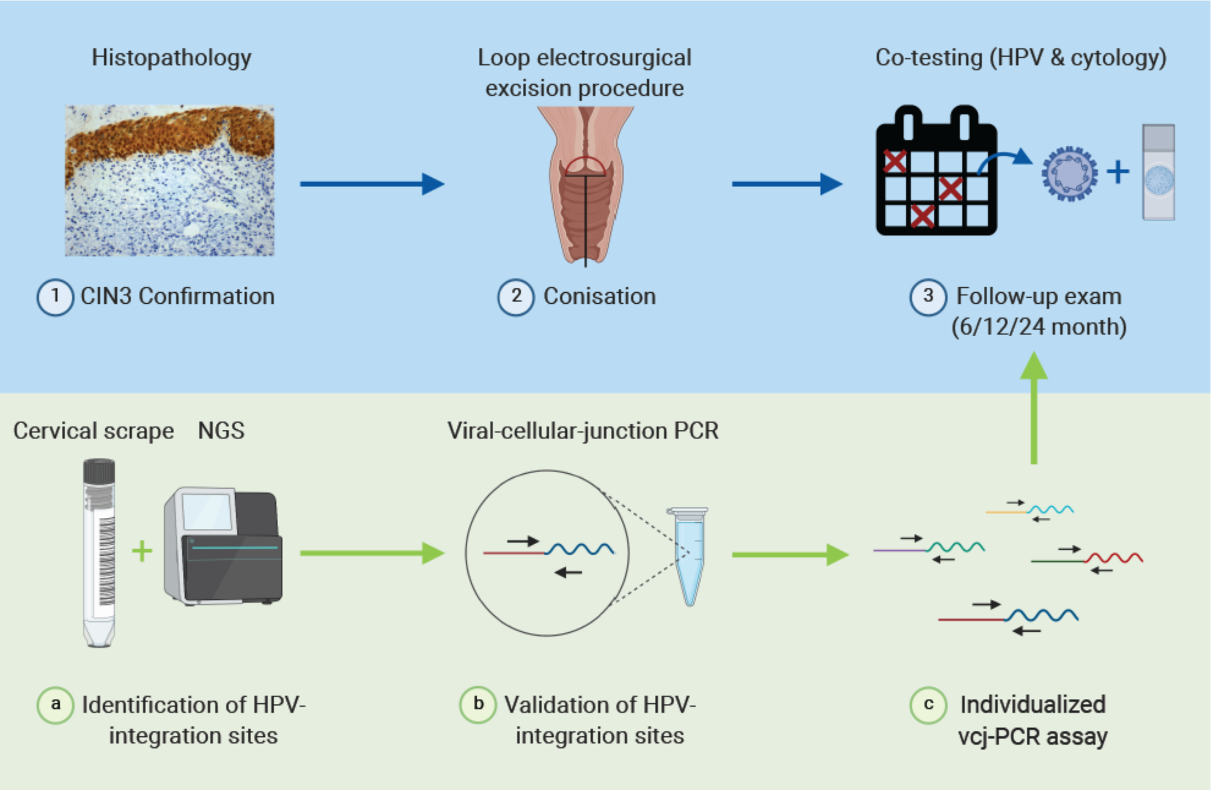

Figure S1: Upper panel depicts disease confirmation by biopsy, surgery and standard follow-up care. Lower panel depicts the steps involved in post-conisation monitoring using an individualized viral-cellular junction PCR
