# Supplementary material for: Evaluation of Integrated HPV DNA as Individualized Biomarkers for the Detection of Recurrent CIN2/3 during Post-Treatment Surveillance
Source: Cancers (Basel). 2021 Jul 1;13(13):3309. doi: 10.3390/cancers13133309 (PMC8269020; doi:10.3390/cancers13133309)
Supplement: Supplementary file 1 [file cancers-13-03309-s001.zip › Supplementary Table S1.pdf]

Table S1: Viral-cellular DNA junctions of all patients validated by vcj-PCR

HPV16 positive

Study ID	Integrat no.	HPV breakpoint[1]	cellular breakpoint	chromosomal map	strand	unique/repetitive cellular sequence	affected gene	targeted region	orientation[2]	overlap (HPV/human) or insertion	Accession number
HPV-03-0002-4[a]	A12-1	2488	68975602	1p31.2	-	u	*AL691520.1*	intron	opposite	0 bp	MZ189169
	A12-2	2897	68999764	1p31.2	-	u	*AL691520.1*	intron	opposite	2 bp overlap	MZ189170
	A12-3	1956	198171457	2q33.1	-	u	*PLCL1*	intron	opposite	2 bp insertion	MZ189171
	A12-4	3312	107132419	3q13.12	-	u	*LINC00882*	intron	same	4 bp overlap	MZ189172
	A12-5	3288	107235101	3q13.12	-	u	*LINC00882*	intron	same	2 bp overlap	MZ189173
HPV-01-0032-2	A154-1	3109	7223812	1p36.23	-	u	*CAMTA1*	intron	opposite	6 bp overlap	MZ189174
	A154-2	2968	22521258	5p14.3	+	u	*CDH12*	intron	opposite	1 bp overlap	MZ189175
	A154-3	3886	126570522	10q26.2	+	u	*C10orf90*	intron	opposite	3 bp insertion	MZ189176
	A154-4	3299	126575535	10q26.2	+	u	*C10orf90*	intron	opposite	6 bp overlap	MZ189177
HPV-03-0225-9	A805-1	2057	63645669	1p31.1	+	u	*PGM1*	intron	same	1 bp overlap	MZ189178
	A805-2	3240	60391808	3p14.2	-	u	*FHIT*	intron	same	1 bp overlap	MZ189179
HPV-05-0019-7[a]	A395-1	3940	203849125	1q32.1	+	u	*ZC3H11A* and *ZBED5*	intron	same	2 bp overlap	MZ189180
	A395-2	2132	203805519	1q32.1	+	u	*ZC3H11A* and *ZBED5*	intron	same	30 bp insertion[d]	MZ189181
HPV-03-0044-0	A69-1	2000	76576678	15q24.3	+	u	*SCAPER*	intron	opposite	0 bp	MZ189182
	A69-2	982	76605022	15q24.3	-	u	*SCAPER*	intron	same	4 bp overlap	MZ189183
HPV-14-0006-0	A184-1	2215	65625366	6q12	+	u	*EYS*	intron	opposite	90 bp insertion[d]	MZ189184
	A184-2	2772	64482049	9q13	-	u	no gene			1 bp overlap	MZ189185
HPV-03-0164-4	A347-1	3209	83012507	2p12	+	u	no gene			9 bp insertion	MZ189186
	A347-2	1995	61587580	14q23.1	+	u	*AL355916.3* and *AL355916.1*	intron	same	3 bp overlap	MZ189187
HPV-10-0006-9	A333-1	2719	49711714	4p11	-	u	no gene			1 bp overlap	MZ189188
	A333-2	2447	23575811	14q11.2	-	u	*JPH4*	exon	opposite	1 bp overlap	MZ189189
HPV-03-0064-6	A89-1	3682	97630788	11q22.1	+	u	no gene			0 bp	MZ189190
	A89-2	4141	[e]			r					MZ189191
HPV-10-0017-5	A306-1	2856	218281931	2q35	-	u	*PNKD* and<br>*TMBIM1*	intron<br>intron	opposite<br>same	5 bp overlap	MZ189192
HPV-01-0061-7	A353-1	2904	1751047	1p36.33	-	r	no gene			3 bp overlap	MZ189193
HPV-15-0008-4	A193-1	3956	194974697	1q31.3	-	u	no gene			0 bp	MZ189194
HPV-07-0011-5	A388-1	2227	92943177	2p11.1	+	r	no gene			1 bp insertion	MZ189195
HPV-01-0078-0	A516-1	3989	71995524	2p13.2	-	u	no gene			3 bp overlap	MZ189196
HPV-10-0042-9[a]	A737-1	3520	17827359	2p24.2	+	u	no gene			3 bp overlap	MZ189197
HPV-10-0020-8	A309-1	2133	60556088	3p14	-	u	*FHIT*	intron	opposite	4 bp insertion	MZ189198
HPV-01-0015-9	A41-1	2454	189892162	3q26	+	u	*TP63*	intron	same	12 bp insertion	MZ189199
HPV-03-0177-6	A404-1	2039	170373504	3q26.2	+	u	*SKIL*	intron	same	3 bp overlap	MZ189200
HPV-12-0024-5[a]	A704-1	2239	169119237	3q26.2	-	u	*MECOM*	intron	same	3 bp overlap	MZ189201
HPV-12-0012-2	A237-1	1044	174513603	3q26.31	-	u	*NAALADL2*	intron	same	24 bp insertion[f]	MZ189202
HPV-03-0082-7	A110-1	3496	189924159	3q28	+	u	*no gene*			1 bp overlap	MZ189203
HPV-12-0007-3	A140-1	3058	196339314	3q29	-	u	*no gene*			1 bp overlap	MZ189204
HPV-03-0078-6[a]	A106-1	3034	57010063	4q12.2	+	u	*POLR2B*	intron	same	3 bp overlap	MZ189205
HPV-10-0010-0	A265-1	2475	73799237	4q13.3	-	u	no gene			3 bp insertion	MZ189206
HPV-03-0160-3	A320-1	2049	111777029	4q25	+	u	no gene			12 bp overlap	MZ189207
HPV-04-0027-6[b]	A422-1	2929	55291554	6p12.1	+	u	no gene			2 bp overlap	MZ189208
HPV-03-0038-5	A63-1	1831	24906146	6p23.2	+	u	*RIPOR2*	intron	opposite	3 bp insertion	MZ189209
HPV-01-0038-9[a]	A170-1	3990	4455663	7p22.2	-	u	no gene			3 bp overlap	MZ189210
HPV-15-0033-9	A553-1	2770	84310279	7q21.11	+	u	*SEMA3A*	intron	opposite	0 bp	MZ189211
HPV-14-0004-4	A148-1	2240	132718508	8p24.22	+	u	*TMEM71*	intron	opposite	4 bp overlap	MZ189212
HPV-10-0015-9	A285-1	2259	108341260	9q31.2	-	u	no gene			3 bp overlap	MZ189213
HPV-07-0044-1	A680-1	3096	128168861	10q26.2	+	u	no gene			2 bp overlap	MZ189214
HPV-15-0026-4	A474-1	3605	8939493	11p15.4	-	u	no gene			5 bp insertion	MZ189215
HPV-12-0003-2	A57-1	3455	102812143	11q22.2	+	u	*WTAPP1*	intron	same	0 bp	MZ189216
HPV-03-0089-7	A117-1	2743	73354925	13q22.1	+	u	no gene			2 bp overlap	MZ189217
HPV-03-0014-7	A23-1	3552	74174461	15q24.1	-	u	*ISLR*	intron	opposite	4 bp overlap	MZ189218
HPV-10-0025-7	A428-1	1075	13143742	16p13.12	-	u	*SHISA 9*	intron	opposite	4 bp overlap	MZ189219
HPV-03-0111-2	A186-1	2691	52327372	16q12.1	-	u	no gene			1 bp overlap	MZ189220
HPV-11-0040-1	A836-1	3303	39425670	17q12	-	u	*MED1*	intron	same	2 bp overlap	MZ189221
HPV-03-0224-1	A806-1	3284	6138989	18p11.31	+	u	*L3MBTL4*	intron	opposite	0 bp	MZ189222
HPV-03-0035-1[a]	A49-1	3699	8480096	19p13.2	-	u	*HNRNPM*	intron	same	4 bp overlap	MZ189223
HPV-15-0039-1	A952-1	3381	1564761	19p13.3	+	u	*MEX3D*	intron	opposite	6 bp overlap	MZ189224
HPV-01-0022-4	A55-1	4019	31729113	20q11.21	-	u	no gene			5 bp insertion	MZ189225
HPV-03-0018-9	A27-1	3500	14975255	20p12.1	+	u	*MACROD2*	intron	same	3 bp insertion	MZ189226
HPV-03-0172-7	A383-1	2877	67605971	Xq12	-	u	*AR*	intron	opposite	13 bp overlap	MZ189227
HPV-03-0109-2	A179-1	1125	90317013	Xq21.31	-	u	no gene			4 bp overlap	MZ189228
HPV-01-0073-1	A470-1	2282	113642153	Xq23	-	u	*XACT*	intron	same	0 bp	MZ189229

HPV18 positive

Study ID	Integrat no.	HPV breakpoint[1]	cellular breakpoint	chromosomal map	strand	unique/repetitive cellular sequence	affected gene	targeted region	orientation[2]	overlap (HPV/human) or insertion	Accession number
HPV-08-0010-5	A273-1	2928	10328807	6p24.3	-	u	no gene			1 bp overlap	MZ189230
	A273-2	3122	101481076	9q31.1	-	u	*PGAP4*	intron	same	2 bp overlap	MZ189231
	A273-3	3720	9757703	11p15.4	-	u	*LINC02709*	intron	same	50 bp insertion[g]	MZ189232
HPV-03-0128-5	A239-1	1589	129878965	7q32.2	+	u	*UBE2H*	intron	opposite	3 bp overlap	MZ189233
	A239-2	2529	33296947	19q13.11	-	u	no gene			3 bp overlap	MZ189234
HPV-10-0008-5	A197-1	3185	125166628	1q12	-	u	no gene			2 bp insertion	MZ189235
HPV-03-0195-1	A596-1	2702	203074561	2q22.3	+	u	*NBEAL1*	intron	same	3 bp overlap	MZ189236
HPV-04-0038-2	A691-1	1909	74035523	4q13.3	+	u	no gene			2 bp overlap	MZ189237
HPV-05-0017-6	A409-1	2705	111899160	6q21	+	u	no gene			19 bp insertion	MZ189238
HPV-01-0089-1	A639-1	1084	4772030	10p15.1	-	u	*AC018978.1*	intron	same	3 bp overlap	MZ189239
HPV-03-0105-5	A161-1	3256	73427645	13q22.1	-	u	*LINC00393*			1 bp overlap	MZ189240
HPV-03-0060-5[c]	A85-1	1669	23369313	15q11.2	-	u	no gene			2 bp insertion	MZ189241
HPV-11-0015-7[a]	A441-1	3448	71549373	15q23	-	u	*THSD4*	intron	opposite	10 bp overlap	MZ189242
HPV-03-0192-8	A599-1	3210	107763776	Xq22.3	-	u	*TSC22D3*	intron	same	7 bp insertion	MZ189243

[1] CIN3 with more than one HPV integrantion site are listed first

[2] Orientation of the cellular gene with regard to the early region of the integrated HPV DNA

[a] CIN3 on biopsy but invasive carcinoma in cone

[b] Patient with CIN3 but excluded because of re-conization

[c] Patient with CIN3 but no surgery

[d] Insert cannot be asigned to any species

[e] sequence can be aligned to multiple chromosomes

[f] HPV DNA nt posn. 1492-1515

[g] HPV DNA nt posn. 2277-2334
